# A period without PER: understanding 24-hour rhythms without classic transcription and translation feedback loops

**DOI:** 10.12688/f1000research.18158.1

**Published:** 2019-04-16

**Authors:** Arthur Millius, Koji L. Ode, Hiroki R. Ueda

**Affiliations:** 1 Laboratory for Synthetic Biology, RIKEN Center for Biosystems Dynamics Research, 1-3 Yamadaoka, Suita, Osaka, 565-0871, Japan; 2 Laboratory of Systems Immunology and Laboratory of Host Defense, Immunology Frontier Research Center, Osaka University, Suita, Osaka, 565-0871, Japan; 3 Department of Systems Pharmacology, Graduate School of Medicine, The University of Tokyo, 7-3-1 Hongo, Bunkyo-ku, Tokyo, 113-0033, Japan

**Keywords:** circadian rhythms, post-transcriptional oscillator, transcriptional-translation feedback loop, red blood cells, dopaminergic ultradian oscillator, peroxiredoxin, phosphorylation

## Abstract

Since Ronald Konopka and Seymour Benzer’s discovery of the gene *Period* in the 1970s, the circadian rhythm field has diligently investigated regulatory mechanisms and intracellular transcriptional and translation feedback loops involving *Period*, and these investigations culminated in a 2017 Nobel Prize in Physiology or Medicine for Michael W. Young, Michael Rosbash, and Jeffrey C. Hall. Although research on 24-hour behavior rhythms started with *Period*, a series of discoveries in the past decade have shown us that post-transcriptional regulation and protein modification, such as phosphorylation and oxidation, are alternatives ways to building a ticking clock.

## Introduction

**Figure 1.**
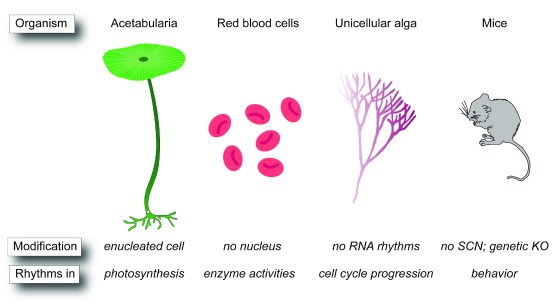
Post-translation oscillators without transcription-translation feedback loops. Examples of post-translation oscillators in enucleated cells such as *Acetabularia* and red blood cells, in unicellular alga lacking RNA rhythms, and in mice in which the classic transcription-translation feedback loop module is disrupted genetically or anatomically. KO, knockout; SCN, suprachiasmatic nucleus.

The time-keeping mechanisms of circadian rhythms can be regulated by multiple layers of different cellular networks, including transcription-translation feedback loops (TTFLs) and post-translation oscillators (PTOs) ^1^. Circadian TTFLs generate oscillations in gene expression through delayed negative feedback whereby expression of a transcription factor negatively regulates its own transcription ^2^. The core of this genetic network in mammals is the expression of a heterodimer of BMAL1 (also called ARNTL) with either CLOCK or NPAS2, which binds at promoter cis-elements called E-boxes to drive expression of genes encoding period (PER1-3), cryptochrome (CRY1-2), and nuclear receptor subfamily (NR1D1-2) proteins, which then repress *Bmal1* expression by a series of separate and interconnected feedback loops ^3, 4^. In contrast to behaviors driven by cyclic differences in gene expression, PTOs generate rhythms independent of transcription and translation through biochemical processes, such as phosphorylation, protein–protein interactions, and other post-translational modifications. These post-translational processes also alter TTFLs as well as post-transcriptional modification of transcripts involved in TTFLs ^5, 6^. The most well-known PTO is the cyanobacteria KaiABC system, which consists of only three proteins and ATP ^7^, but novel PTOs may also exist in red blood cells (RBCs) ^8– 14^, which lack a nucleus and the molecular machinery to drive TTFL rhythms. In addition, a series of new and old observations of 24-hour rhythms in biological contexts where classic TTFLs are absent or diminished ( Figure 1) ^15– 23^ continue to puzzle researchers and demonstrate that there are multiple ways to build a clock.

One of the first modern uses of the term “circadian” was to describe 24-hour endogenous oscillators that alter *Drosophila* fly behavior rhythms ^24^, and the persistence of oscillations at various temperatures was viewed as a defining feature of circadian rhythms ^25, 26^. The first genetic component of circadian rhythms was discovered in the 1970s when Ronald Konopka in Seymour Benzer’s lab used chemical mutagenesis of *Drosophila* to discover three alleles of the *Period* gene ^27^. In the 1980s, rhythmicity of *Period* mutants was shown to be restored by gene transfer ^28, 29^, confirming that *Period* both is necessary and can restore rhythmic behaviors, such as eclosion and locomotor activity, in flies. In 1990, Hardin *et al*. proposed that PER protein altered the levels of *Period* mRNA in a negative feedback loop ^30^, but at the time it was unclear whether PER directly suppressed *Period* transcription or whether the negative feedback occurred through an indirect route. A few years later, researchers discovered that this negative feedback was direct in the bread mold *Neurospora crassa* model of circadian rhythms because the frequency (FRQ) directly repressed its own transcription ^31^. In addition to *Neurospora* ^32^ and *Drosophila* ^33^, TTFL models of circadian rhythms from plants ^34^ to mammals ^35^ have been elucidated and reviewed extensively.

## Post-translational oscillators and post-translational modifications: breaking the transcription-translation feedback loop mold

The modern idea that TTFLs were necessary for 24-hour rhythms was shattered in 2005 when Nakajima *et al*. reconstituted rhythmic 24-hour oscillations in protein phosphorylation with just a small number of cyanobacterial proteins ^36^. This seminal moment in the circadian rhythm field spurred investigators to examine other non-canonical rhythm-generating mechanisms and to unearth forgotten studies of PTOs. For example, in the 1960s, it was shown that the unicellular alga *Acetabularia* undergoes diurnal rhythms of photosynthesis, which persist even after the nucleus has been artificially removed ^21^.

There are a few more recent examples of organisms that have circadian rhythms in the absence of TTFLs. In the unicellular red alga *Cyanidioschyzon merolae*, circadian rhythms control cell cycle progression in the absence of RNA translation ^22^, and the unicellular dinoflagellate *Lingulodinium* has daily rhythms in bioluminescence and photosynthesis without a detectable change in RNA transcript abundance and in the presence of transcription inhibitors ^23^. These studies suggest that protein activities and post-translational modifications can serve as 24-hour oscillators. Research has centered on phosphorylation as the period-determining post-translational modification ^37– 40^, but other post-translational modifications, including methylation, acetylation, sumoylation, and ubiquitination, also alter clock function ^41– 44^.

Importantly, circadian rhythms are insensitive to temperature and this property of temperature compensation was identified in biological time-keeping systems, such as those of bees, flies, and marine organisms, as early as the 1950s and 1960s ^25, 26, 45, 46^. Transcription and translation are temperature-dependent reactions ^47– 50^, which suggests that post-translational activities are important for temperature compensation. For example, Isojima *et al*. revealed that phosphorylation by casein kinase I (CKI) is a temperature-insensitive period-determining process, and the degradation rate of PER2, which is regulated by CKI phosphorylation, was found to be insensitive to temperature ^38^. Importantly, the phosphorylation of PER2-derived peptide by CKI is insensitive to temperature *in vitro*. In 2015, the degradation of PER2 was found to occur in a more complex mode composed of three distinct stages, and the duration of the second stage depended on circadian time, which led to the identification of temperature-sensitive and -insensitive PER2 phosphorylation sites ^51^. Thus, differences in the temperature sensitivity of phosphorylation sites on the repressor, which alter degradation rates at different temperatures, are responsible for temperature compensation, PER2 stability, and ultimately the length of the circadian period. In 2017, Shinohara *et al*. identified a short sequence region around residue K224 in CKI, which was responsible for temperature compensation and converted a temperature-sensitive kinase into a temperature-insensitive one *in vitro* ^52^. Mutation of K224 shortens circadian behavioral rhythms and alters the temperature dependency of the circadian clock in the sub-hypothalamic region of the brain ^52^, called the suprachiasmatic nucleus (SCN), which controls circadian response to light. It is though noteworthy that K224 is part of the consensus KRQK monopartite nuclear localization signal in CKI, which makes it difficult to disentangle the effects of temperature dependence from that of localization *in vivo*. These studies provide evidence for how post-translational activities modify TTFL rhythms, but a series of new and old studies have revealed that PTOs can drive rhythms even in the absence of TTFL clocks.

## Blood: a novel source of post-translational oscillator rhythms

Mammals have a natural supply of enucleated cells in RBCs, and researchers have plumbed this cell type for non-TTFL rhythms. In the 1970s, circadian rhythms in ATPase activity and periodic rhythms in enzymes—such as acetylcholinesterase, glyceraldehyde-3-phosphate dehydrogenase, and glucose-6-phosphate dehydrogenase—in RBCs were found ( Table 1), but it was unclear whether the rhythms were robust or persistent beyond 24 hours ^10^.

**Table 1.** Oscillatory phenomena observed in human red blood cells.

Molecule	Year	Period	Impact	Reference
Glucose-6-phosphate dehydrogenase	1975	~12 hours	Observed two peaks in enzyme activity over a 24-hour period in three different individuals	13
Glutamate oxaloacetate transaminase	1975	~12 hours	Observed two peaks in enzyme activity over a 24-hour period in two different individuals	13
Acid phosphatase	1975	~24 hours	Observed one peak in enzyme activity over a 24-hour period in one individual in plasma-free human red blood cell suspensions	13
Acetylcholinesterase	1975	~24 hours	Observed one peak in enzyme activity over a 24-hour period in two individuals	13
Glucose-6-phosphate dehydrogenase	1976	~12 hours	Observed two peaks in activity over a 24-hour period with one pattern peaking at 4 p.m. and midnight and the other peaking at midnight and 8 p.m. in six and five individuals, respectively	14
6-phophogluconate dehydrogenase	1976	~12 hours	Observed two peaks in activity over a 24-hour period peaking at 4 a.m. and 4 p.m. in 11 individuals	14
Lactic dehydrogenase	1976	~12 hours	Observed two peaks in activity over a 24-hour period with one pattern peaking at noon and midnight and the other peaking at 4 a.m. and 4 p.m. in four and seven individuals, respectively	14
Aspirate aminotransferase	1976	~12 hours	Observed two peaks in activity over a 24-hour period peaking at 4 a.m. and 4 p.m. in 11 individuals	14
Hexokinase	1976	~24 hours	Observed one peak in activity over a 24-hour period peaking at 4 p.m. in 11 individuals	14
Potassium efflux	1976	NS	Observed a steady increase in potassium efflux over a 48-hour period in an unknown number of individuals (averaged data reported)	12
Membrane potential	1976	~24 hours	Observed two peaks in membrane potential by DiOC _5_(3) over a 48-hour period in an unknown number of individuals (averaged data reported)	12
Mg-dependent ATPase	1976	~24 hours	Observed one peak in activity from human blood bank bags incubated at 37 °C for 27 hours (average of eight samples)	8
Acetylcholinesterase	1978	NS	Observed variations in acetylcholinesterase activity over a 24-hour period in four individuals, but variations had lower amplitude than reference ^13^ and were not circadian	10
Peroxiredoxin	2011	~24 hours	Observed three peaks of peroxiredoxin dimer oxidation and PRX-SO _2/3_ abundance over a 60-hour period in three individuals	11
NADH	2011	~24 hours	Observed three peaks in NADH abundance over a 60-hour period in three individuals	11
NADPH	2011	~24 hours	Observed three peaks in NADPH abundance over a 60-hour period in three individuals	11
Membrane potential	2017	~24 hours	Observed two peaks in membrane potential by dielectrophoresis, DiOC _5_(3), and mass spectrometry over a 48-hour period in four biological replicates	9
Membrane conductance and cytoplasm conductivity	2017	~24 hours	Observed two or three peaks in membrane conductance and cytoplasm conductivity over a 48-hour period in four individuals	9
Intracellular potassium	2017	~24 hours	Observed two peaks in intracellular potassium concentrations over a 48-hour period in four biological replicates	9

NS, not significant.

In 2011, an anti-oxidant enzyme called peroxiredoxin (PRX) in cultured human RBCs was found to have temperature-independent circadian cycles of hyperoxidation for up to 76 hours ^11^. Because RBCs lack a nucleus and the rhythms persisted in the presence of transcription and translation inhibitors, a novel non-transcriptional-based circadian oscillator in mammals was proposed. Analysis of the PRX rhythms relied solely on PRX1, PRX2, and PRX-SO _2/3_ (hyperoxidized PRX form) antibodies. In particular, the PRX-SO _2/3_ antibody recognizes multiple hyperoxidized forms of PRX ^53^, results in up to eight different bands on non-reducing sodium dodecyl sulfate–polyacrylamide gel electrophoresis (SDS-PAGE) ^11^, and produces multiple non-specific bands that can confound interpretation of the hyperoxidized signal ^54, 55^, which make determination of the correct PRX isoform technically difficult and in-gel controls essential. Nevertheless, the same researchers discovered that hyperoxidized PRX-SO _2/3_ rhythms were conserved in a wide range of species ^55, 56^.

In mice, blocking hemoglobin oxygen transport by incubation with carbon monoxide eliminates PRX2 hyperoxidized rhythms ^57^. Hemoglobin auto-oxidation in RBCs generates superoxide, which is converted to H _2_O _2_ by superoxide dismutase 1 (SOD1) ^58, 59^, and H _2_O _2_ is subsequently reduced by catalase, glutathione peroxidase, and PRXs ^54, 58, 60^, which results in the oxidation of these proteins ^61^. Oxidation of PRX2 is reversed by sulfiredoxin (SRX) ^62– 64^, but rhythms in PRX2 oxidation in mice are not mediated by the rhythmic reduction of hyperoxidized PRX2 by SRX but rather through rhythmic degradation by 20S proteasomes, and only about 1% of the total PRX pool is modified in a circadian manner ^57^. Mitochondria-specific PRX (PRX3) is also reversibly inactivated by hyperoxidation, reduced, and reactivated by SRX, and hyperoxidized PRX3 and SRX undergo anti-phasic circadian oscillations in the mitochondria in various mice tissues, which links mitochondria function to circadian rhythms ^65^. Another group revealed about three peaks in hyperoxidized PRX-SO _2/3_ rhythms in mice over a 48-hour period (instead of two as would be expected for a circadian rhythm) and showed that rhythms were impaired in SOD1-mutant mice ^66^. There is still uncertainty regarding the origin of PRX hyperoxidation rhythms, but the data suggest that PRX-SO _2/3_ oscillations are more an output of rhythm-generating machinery involving the 20S proteasome rather than a daily oxidation-reduction cycle.

Deconstructing PRX rhythms biochemically and using non-antibody methods, such as mass spectrometry, to directly detect the hyperoxidized cysteine residue or redox-sensitive fluorescent proteins may bolster understanding of this novel PTO. However, biochemical reconstruction is difficult because RBC lysis causes gradual loss of PRX-SO _2/3_ signal over a 48-hour period ^9^. However, these types of approaches have revealed that potassium-containing media enhances PRX2-SO _2/3_ rhythms ^9^, and chemical perturbation with Conoidin A, a PRX2 inhibitor ^67^, shortens PER2:LUCIFERASE rhythms in immortalized mouse fibroblasts ^68^.

The exact mechanism of rhythmic PRX oxidation is still unclear, but researchers have begun to examine other general rhythmic behaviors in RBCs. Although no one has followed up on the circadian, ultradian, and irregular rhythms of various enzyme activities in the 1970s, in 2011 researchers reported circadian changes in NADH and NADPH levels ^11^, and in 2017 researchers reproduced circadian changes in RBC membrane potential ^9^ observed in an article published in 1976 ^12^. Paradoxically, the researchers reported circadian changes in potassium concentration in 2017, whereas no rhythms in potassium were observed in 1976; instead, a gradual and steady increase in potassium efflux occurred over the 48-hour observation period. Whether these differences arise from measuring slightly different potassium populations (intracellular versus extracellular), a small sample size, technical differences in methods and individuals, or an actual biological phenomenon remains to be determined.

## Post-translation control of circadian period in transcription-translation feedback loop model organisms

There are shared design principles between the period-determination processes of PTO-based and TTFL-based oscillators. A PTO generates rhythmic changes in protein *states* without changing the amount of protein itself. On the other hand, rhythmic protein synthesis and degradation are essential for TTFL-based oscillations, and mechanisms that control protein *abundance* are critical for controlling the circadian period. This idea is widely accepted for circadian TTFL oscillators because there is a significant correlation between the half-life of transcription repressor mutants, such as in *Drosophila* PER and *Neurospora* FRQ, and circadian period ^69, 70^. The correlation suggests that faster degradation of circadian repressors accelerates clock speed. In mammalian circadian clocks, F-box proteins recruit E3 ubiquitin ligase complexes that license PER and CRY degradation, which modulates period length ^71^. Although the circadian TTFL-based oscillators involve post-translational regulation as period-determination mechanisms, modification of transcription repressors regulates period length by changing repressor stability. For example, a mutation in CKIε that destabilizes mammalian PER results in period shortening ^72, 73^, and mutation of a phosphorylation site on PER that destabilizes PER also results in period shortening ^74, 75^. Other kinases, such as AMPK and DNA-PK, control period length by altering CRY stability through phosphorylation ^76, 77^. In addition, stabilization of CRY by small molecules lengthens the period ^78^, and destabilization of CRY by degron tagging of CRY shortens the period ^79^, strongly suggesting the causal relationship between CRY stability and period length.

However, a recent study of the *Neurospora* circadian clock challenged this protein stability–period length paradigm of period determination in a TTFL-based oscillator ^80^. Researchers used an FWD-1–deleted strain, which is an F-box protein that causes proteolysis of phosphorylated FRQ. The *Δfrd*-1 strain results in a markedly increased FRQ half-life, and new FRQ is produced even in the presence of hyperphosphorylated FRQ. Nonetheless, circadian oscillation of FRQ-promoter activities persists with modest change in period length, and several short-period mutations of FRQ still have a short period in a *Δfrd*-1 background in which the stability of FRQ is significantly increased. Because mutation of phosphorylation sites in FRQ still alters the period and because generic inhibition of kinase activity lengthens the period even in the absence of FWD-1, these data suggest that a protein-state not a protein-abundance attribute, namely phosphorylation, controls period length.

A similar uncoupling of protein stability and circadian period may occur even in the TTFL clock in mammals. A recent study of CRY1 mutations in phosphorylation sites by Ode *et al*. revealed that multiple phosphorylation sites near the co-factor binding pocket of CRY1 markedly changes period length while having only a modest effect on CRY1 half-life ^79^. Mutagenesis of CRY1 and CRY2 revealed mutations in a secondary co-factor binding pocket which shorten the period without reducing CRY1 stability ^81^. Furthermore, an exon-skipping mutation in CRY1 found from a human family with delayed sleep phase syndrome lengthens the period without affecting CRY1 stability ^82^. Therefore, mammalian CRY may also control the circadian period independently of its abundance.

If protein abundance control does not explain all aspects of period determination, what is the nature of state control of TTFL-based oscillator proteins such as multisite phosphorylation of FRQ, PER, and CRY? One of the shared properties of period-determining repressor proteins is structural flexibility. Most FRQ and PER regions modified by multisite phosphorylation are intrinsically disordered, highly flexible, and variable ^83, 84^. The multisite phosphorylation region of CRY1 critical for period control also occurs on a flexible loop region. These flexible regions may undergo a relatively large conformation change that may underlie slow dynamics (that is, 24 hours) of protein activity change. The intrinsically disordered C-terminal domain of BMAL1 controls the period through a slow conformation change with a high energy barrier ^85^. Conformation changes may lead to a slow and coherent re-organization of the macromolecular repressor complex ^86^, which is consistent with the dynamics of the cyanobacteria PTO ^87, 88^ in which the slow dynamics of the intrinsic conformational change of KaiC ^89^ couple to the re-organization of the KaiABC complex ^90^. An atomic-scale understanding of the repressor complex in a TTFL-based oscillator may reveal subtle differences in molecular mechanisms of 24-hour period determination between PTO- and TTFL-based oscillators.

## Oscillations without classic transcription-translation feedback loop oscillators

Several classic models of circadian rhythms have persistent 24-hour rhythms even when the circadian TTFL machinery is absent or disrupted. In S2 cells, which are generally regarded as non-rhythmic, a multi-omics approach recently revealed hundreds of genes, proteins, and metabolites with 24-hour rhythms ^20^. Although this approach seems to suggest the presence of a novel non-canonical oscillator with 24-hour periodicity, it does not preclude possible cell cycle effects from the roughly 24-hour doubling time of S2 cells or the possibility of classic circadian clock components operating below the experimental limits of detection. For example, large-scale proteomics studies of circadian variation frequently fail to detect circadian proteins ^91, 92^ because there may be only a few hundred to a thousand protein copies per cell ^93^. Thus, genetic knockout (KO) of canonical clock genes is needed to definitively determine whether rhythms derive from a novel oscillator.

In mammals, genetic and anatomical ablation of the circadian machinery normally disrupts 24-hour behavioral rhythms, but rhythms persist under specialized situations. For example, SCN-lesioned rats administered methamphetamine in the drinking water retain circadian behaviors of activity in constant light conditions ^17^. This so-called methamphetamine-sensitive oscillator also does not depend on classic circadian genes, such as *Per1-2*, *Cry1-2*, *Bmal1*, *Npas2*, and *Clock* ^18, 94^. Recent data suggest that the methamphetamine-sensitive oscillator is a long-period manifestation of a tunable dopamine ultradian oscillator ^95, 96^. KO of a dopamine transporter in SCN-lesioned or *Bmal1* KO mice, which prevents dopamine reuptake in dopaminergic neurons, increases the period of the ultradian rhythms. Similarly, administration of methamphetamine, which increases extracellular dopamine concentrations, lengthens ultradian rhythms in a dose-dependent manner from 4 hours to an astonishing 48 hours. In contrast, the anti-psychotic drug haloperidol, which selectively blocks the dopamine D2 receptor, shortens long-period rhythms induced by methamphetamine in wild-type and *Bmal1* KO mice ^95^. These data suggest that dopamine neurons are a second independent rhythm-generating mechanism in the brain, and future studies using chemical and genetic approaches to perturb dopamine pathways coupled with recently developed brain-clearing techniques ^97– 100^ may enable a more complete understanding of the neural architecture of this dopamine ultradian oscillator.

## Conclusions

From blood to brain, these studies suggest that non-canonical PTOs have an impact on circadian rhythms beyond the classic PER negative feedback loop. However, recent studies of PER itself, including temperature-sensitive phosphorylation sites ^51^, three prime untranslated region (3′-UTR) regulation ^101^, and the separation of *Period2* rhythms from *Bmal1* rhythms in the SCN ^102^, indicate that even a gene as well studied as *Period* can still teach us new tricks about the period-determining mechanisms of circadian rhythms.

## Abbreviations

AMPK, AMP-activated protein kinase; BMAL1, brain and muscle Arnt-like protein 1; CKI, casein kinase I; CLOCK, circadian locomotor output cycles kaput; CRY1-2, cryptochrome1-2; FRQ, frequency; FWD-1, F-box/WD-40-repeat-containing protein 1; KO, knockout; NPAS2, neuronal PAS domain-containing protein 2; NR1D1-2; nuclear receptor subfamily 1 group D member 1-2; PER; period; PRX, peroxiredoxin; PTO, post-translational oscillator; RBC, red blood cell; S2, Schneider 2; SCN, suprachiasmatic nucleus; SOD1, superoxide dismutase 1; SRX, sulfiredoxin; TTFL, transcription-translation feedback loop.
